# Commentary: “Compensatory plasticity: time matters”

**DOI:** 10.3389/fnins.2015.00348

**Published:** 2015-10-08

**Authors:** Nuno M. Gama, Alexandre Lehmann

**Affiliations:** ^1^International Laboratory for Brain, Music and Sound Research, Center for Research on Brain, Language and MusicMontreal, QC, Canada; ^2^Medical Research Council Institute of Hearing ResearchNottingham, UK; ^3^Department of Otolaryngology Head and Neck Surgery, McGill UniversityMontreal, QC, Canada

**Keywords:** cochlear implants, brain plasticity, critical period, early implantation, deprivation

The mammalian nervous system can adapt to the challenges of life through neural plasticity. The brain will undergo extensive reorganization following sensory deprivation or damage to afferent pathways (Kaas, 2001). This plastic reorganization develops as a function of time. A recent review on plasticity in the blind (Lazzouni and Lepore, 2014) stressed the importance of critical periods and the influence of the duration of sensory deprivation on the re-organization of sensory cortices. Such considerations are paralleled in the hearing sciences, sharing the authors' opinion that “time matters.” Restoring lost sensory function to a blind or deaf cortex, via surgically implanted devices offers a unique insight into brain reorganization, allowing scientists to follow the transition from deaf to hearing, from blind to sighted. While retinal implants are just becoming available in a clinical setting (Zrenner et al., 2010), cochlear implants (CIs) have been offered since the 1980s (Clark, 2003). Here we argue that, for auditory implants, time matters along two dimensions: pre and post-implantation. On the one hand, plasticity is especially strong when sensory deprivation occurs at early stages of development. Referred to as the sensitive period for brain development, it provides cut-off ages to guide implantation (Sharma et al., 2002; Bedny et al., 2010). On the other hand, the functional maturity of the auditory cortex crucially depends on sensory experience (Kral et al., 2005), emphasizing the importance of rehabilitation.

Age at implantation plays a substantial role in performance with a CI. Research has shown the existence of an early critical period for brain development and demonstrated how deprivation-driven functional changes in the cortex are affected by age. Cats that were implanted after the fifth month of age had smaller activation areas of the auditory cortex, compared with cats implanted earlier, even when they had longer experience with implant (Kral et al., 2002, 2005; Kral and Sharma, 2012).

In humans, the latency and morphology of the P1 component of auditory-evoked potentials can serve as a biomarker for the development of the central auditory pathways (Sharma et al., 2005b; Dorman et al., 2007; Kral and O'Donoghue, 2010). Using this measure, a cut-off age for optimal auditory cortical plasticity was identified. Children implanted before the age of 3.5 years showed a faster and more robust development of the P1 than children implanted past age seven. Sharma et al. (2002) observed that 55 out of 57 early-implanted children had P1 latencies within the range of age-matched normal-hearing children vs. 10 out of 29 middle-implanted and 1 out of 21 late-implanted children, despite all children being matched for implant use duration. In a longitudinal study, late-implanted children showed atypical P1 latencies and morphologies during the first year of implantation whereas the early-implanted group showed a more rapid development (Sharma et al., 2005a). Behavioral studies also show faster and better language development in children implanted before the age of 3 (Nikolopoulos et al., 1999; Kirk et al., 2002), which correlates well with functional changes in the brain (Lee et al., 2001; Giraud et al., 2002). Thus, both animal and human CI studies suggest that age at implantation has a stronger influence on performance than duration of experience with the implant.

Despite the dominant role of age at implantation, experience with a CI also induces functional changes in the auditory system—at both peripheral and central level—stressing the importance of a post-implantation rehabilitation period (Sandmann et al., 2009). Spiral ganglion neurons play a critical role in relaying the afferent auditory information; studies in deafened cats and guinea pigs showed that electrical stimulation prevented their retrograde degeneration and increased their size (Shepherd et al., 1983, 1994; Losteau, 1987; Leake et al., 1991, 1999; Li et al., 1999). Similarly, electrical stimulation through CIs had a restorative effect on the medial superior olive, a key auditory brainstem structure (Tirko and Ryugo, 2012). In humans, electrical stimulation resulted in functional improvements. Activation of both primary and secondary regions of the auditory cortex in response to sound was observed in CI patients 1 week after implant switch-on (Giraud et al., 2001); as CI experience increased, the authors consistently observed a reduction in the number of activated clusters in the secondary regions, indicating a better tuning of primary auditory region. This effect was smaller in late-implanted than in early-implanted individuals, illustrating the complex interaction between the factors of implant experience and age at implantation (Giraud et al., 2001; Kral et al., 2002).

Returning to the parallel between visual and auditory deprivation, studies on critical period and visual implantation are still not possible as retinal implants are currently only available to adults. However, several questions arise, for instance, can we observe a similar benefit when implanting during the sensitive period for retinal implants? As hearing aid use correlates with positive CI outcomes (Lazard et al., 2012), will the use of sensory substitution devices in the blind benefit or hinder visual restoration? On par with Lazzouni and Lepore's review (Lazzouni and Lepore, 2014), research on auditory deprivation supports the compensatory adaptation theory, whereby the deafferentiated cortex reorganizes and adapts to sensory loss. Anatomical and functional changes take place in the auditory-deprived brain, contrary to the general loss hypothesis that would predict undifferentiated degradation of sensory function. Moreover, research on CIs provides a different perspective on the compensatory adaptation theory. Whether this reorganization is detrimental or beneficial to hearing with a CI is very much an open debate (see Heimler et al., 2014 for a review). Cross-modal reorganization of the auditory cortex can limit the benefit from a CI (see for instance Sandmann et al., 2012), but in other cases, it can enhance it (Mitchell and Maslin, 2007; Rouger et al., 2007). Time spent in the deprived state may play a key role in solving this apparent contradiction (Giraud and Lee, 2007; Lee et al., 2007). How soon is too soon for surgical implantation and activation to maximize the risk-benefit trade off is debated and highly depends on individual factors (James and Papsin, 2004; Colletti et al., 2012; Hagr et al., 2015).

In sum, both the time spent in a deprived state and acquiring sensory experiences matter for the brain to cope with the challenges of change. Stimulating the impaired sense as much as possible and restoring it as soon as possible within the cut-off period shall maximize the benefits from implantation.

## Conflict of interest statement

The authors declare that the research was conducted in the absence of any commercial or financial relationships that could be construed as a potential conflict of interest.
